# Editorial: Intra/Extracellular Dynamics of the Respiratory System and Global Airway Disease

**DOI:** 10.3389/fcell.2020.00523

**Published:** 2020-07-09

**Authors:** Wei-jie Guan, Thai Tran, De-Yun Wang

**Affiliations:** ^1^State Key Laboratory of Respiratory Disease, National Clinical Research Center for Respiratory Disease, Guangzhou Institute of Respiratory Health, First Affiliated Hospital of Guangzhou Medical University, Guangzhou Medical University, Guangzhou, China; ^2^Department of Physiology, Yong Loo Lin School of Medicine, National University of Singapore, Singapore, Singapore; ^3^Department of Otolaryngology, Yong Loo Lin School of Medicine, National University of Singapore, Singapore, Singapore

**Keywords:** barrier function, airway remodeling, smooth muscle, asthma, epithelial proliferation

Chronic inflammatory airway diseases are a heterogeneous entity that encompasses an extensive array of allergic (i.e., allergic rhinitis, asthma, eosinophilic bronchitis) and non-allergic diseases (i.e., chronic rhinosinusitis, chronic obstructive pulmonary disease, bronchiectasis, cystic fibrosis). The concept “one airway, one disease” has been increasingly accepted thanks to the accumulating evidence that has pointed to the association between upper and lower airway diseases. Hence, the term “global airway disease” or “united airway disease” might more accurately reflect the co-existing diseases as well as the potential interactions between upper and lower airway diseases (Burgel, 2015; Giavina-Bianchi et al., 2016). Notwithstanding our growing recognition of this concept, there remain many unanswered questions. What are the common triggers of global airway diseases and how do they work? What are the mechanisms underlying these associations? What are the potential therapeutic targets for intervention? In this special issue of *Frontiers in Cell and Developmental Biology*, a series of original research and review articles have sought to unravel some novel mechanistic insights as well as potential therapeutic targets of the global airway diseases.

A number of mechanisms related to the global airway disease have been proposed. Central to these mechanisms is the contribution of the epithelium. Lining both the upper and lower airways, the airway epithelium constitutes a critical layer barrier against the invasion of pathogens, antigens, and other xenobiotics. Thus, disruption of the airway epithelial barrier function has been associated with the pathogenesis of various airway inflammatory diseases. In a review by Laulajainen-Hongisto et al., the authors elegantly summarized that the pathogenic factors contribute to the weakening of airway epithelial barrier function by disrupting the tight junction (i.e., airborne irritants and pollutants) and mucociliary function (i.e., viral infections, aeroallergen inhalation) and shaping the immune responses (i.e., dysbiosis). Respiratory viral infections (in particular, latent infections) disrupt mucociliary clearance, mucus hypersecretion and epithelial cell death, which further readily result in airway dysbiosis, cellular and tissue oxidative stress and the dysregulation of inflammatory responses (Tan K. S. et al.). Similar with viral infections, air pollutants (especially particulate matter) result in decreased mucociliary clearance, increased pathogen adhesion, airway dysbiosis, decreased alveolar macrophage and natural killer cells, and decreased antimicrobial activity—all of which can compromise factors epithelial barrier function (Yang et al.).

Apart from the defective epithelial barrier function, certain molecules that are constitutively expressed also in the upper and lower airways play key roles in modulating the development of global airway diseases. For instance, cluster of differentiation 151 (CD151) facilitates viral replication via nuclear export signaling and up-regulation of CD151 contributes to airway hyperresponsiveness and smooth muscle contractility (hallmarks of asthma), and lung fibrosis via modulating matrix metalloprotein-7 expression (Wong and Tran). In addition, cell senescence as a consequence of telomere damage, oxidative stress, cellular inflammation, and/or non-selective autophagy, triggered by various pathogenic factors, might also underlie the pathogenesis of global allergic diseases such as asthma (Wang Z.-N. et al.). Moreover, airway smooth muscle cells are vulnerable to inflammation triggers (such as exaggerated release of tumor necrosis factor-α and interleukin-13) which could lead to smooth muscle dysfunction that is related to endoplasmic reticulum stress and mitochondrial dysfunction (Delmotte and Sieck). In another study, the interaction between A-kinase anchoring proteins and phosphokinase A was capable of inducing smooth muscle hypercontractility with concomitant induction of cell proliferation markers (Baarsma et al.). All these novel findings provide further insight into the molecular mechanisms underlying smooth muscle dysfunction that cannot be readily ameliorated by the use of bronchodilators. Collectively, an integrated approach for the disease severity assessment and clinical management is warranted in light of the multifaceted nature of global airway diseases.

Apart from the adverse impacts conferred by direct exposure after birth, maternal exposure to air pollution and cigarette smoke could lead to epigenomic reprogramming leading to altered immune response and aberrant epithelial and mesenchymal cellular function of the fetus that underpin the development of asthma during childhood (Wang B. et al.). The accumulating evidence has pointed to the potential benefits of cessation of cigarette smoking and initiating campaigns to improve air quality to mitigate the risks of allergic diseases such as asthma. Finally, physical factors (such as irradiation) have also been shown to disrupt barrier function by causing cell shape elongation and increasing cell motility—both of which contribute to cell unjamming transition which was dependent on transforming growth factor-β (O'Sullivan et al.). The development of global airway diseases is, therefore, likely to be associated with cellular epigenomic reprogramming and defective epithelial barrier function (i.e., alteration in cell proliferation and motility).

Yet the impact of certain global airway diseases might be more far-reaching. An anecdotal finding of the sequelae of decreased olfactory sensitivity (possibly as a result of allergic rhinitis or chronic rhinosinusitis) was the association with erectile dysfunction. In a prospective study of 102 males, Deng et al. observed a significant correlation between the olfactory function and erectile function. Interestingly, after adjustment for potential confounding factors, erectile dysfunction was associated with the olfactory disorder, rhinology disease, age, and greater visual analog scale (a 5-point scale self-rated by the patients, with higher scores indicating a greater magnitude of symptom burden) for olfactory function (Deng et al.). Therefore, inflammatory upper airway diseases might have more profound systemic implications, although further mechanistic studies are warranted.

We have also had the privilege of spotting some novel targets that would help manage the global airway diseases in the near future. Asthma is one of the most common airway inflammatory diseases which frequently co-exist with allergic rhinitis or chronic rhinosinusitis. Maintaining proper immune balance is crucial to asthma management. Allergen immunotherapy has been effective in a subset of patients with allergic rhinitis or asthma, but the specific mechanisms are not fully elucidated. Feng et al. revealed that an increase in the level of specific immunoglobin G4, but not specific immunoglobin E, against *Dermatophagoides pteronyssinus* correlated with the magnitude of amelioration of basophil activity after subcutaneous allergen immunotherapy. The competence of specific immunoglobin G4 antibodies with specific immunoglobin E binding to the allergens helped interpret why allergen immunotherapy worked at least in certain subpopulations (Feng et al.). These findings represented an opportunity to refine the clinical efficacy of allergen immunotherapy. Therapeutic approaches that could boost the suppression of basophil activation via potent binding with the allergens would be an important adjunct to allergen immunotherapy.

Similar to the vicious cycle seen in airway suppurative diseases, cellular senescence might further result in accelerated aging of the neighboring cells (Wang Z.-N. et al.). Targeted interventions (i.e., caloric restriction, supplementation of antioxidants, senolytic drugs) and replenishment of cells (i.e., stem cell transplantation) might have a role in averting the accelerated senescence of cells and tissues as a consequence of inflammatory insults.

Airway remodeling has been regarded as the hallmark of many chronic airway inflammatory diseases such as asthma and chronic obstructive pulmonary disease. While epithelial membrane thickening and smooth muscle hyperplasia have been identified to be the predominant changes, few studies have linked these changes to the molecular mechanisms. Tan Y. et al. elaborated on the role of fibrocyte growth factor-2 (FGF-2), a potent mitogenic factor, in modulating immunity against viral infections and chronic airway inflammation. Being the “relay player” between airway structural cells (which sensed the insults) and inflammatory cells (usually the effector cells), FGF-2 could promote neutrophil recruitment, activate monocytes, induce smooth muscle cell hyperplasia, and promote its release of cytokines. These have rendered FGF-2 a potentially promising target for future therapeutic interventions that aim at ameliorating airway remodeling.

Bronchiectasis is a chronic airway suppurative disease characterized by irreversible dilatation of bronchi and bronchioles. Through immunofluorescence assays on the surgically resected specimens, Peng et al. have identified the presence of cuboidal and columnar epithelial hyperplasia with disarrangement of thyroid transcription factor-1 (TTF-1)^+^ cells in patients with bronchiectasis. Importantly, most progenitor cell markers (Clara Cell 10, surfactant protein C and p63) co-localized with TTF-1 in the dilated bronchiole sub-epithelium. Expression of surfactant protein C co-localized with TTF-1 in regions with cuboidal epithelial hyperplasia, whereas TTF-1 co-localized with P63 and surfactant protein C in regions with columnar epithelial hyperplasia. These pilot studies link the abnormality of airway progenitor cells to the pathogenesis of bronchiectasis, thus unraveling the therapeutic targets for bronchiectasis.

Figure 1 summarizes the emerging understandings of the causes of the diseases, molecular mechanisms, and possible targets for therapy. We are now in a better position to appreciate the molecular mechanisms responsible for the tissue and cellular injury. A systemic approach (which takes into account the epithelial barrier function, airway remodeling, immune dysregulation, oxidative stress, and dysbiosis) is needed for an integrated clinical assessment and management of the global airway diseases.

**Figure 1:**
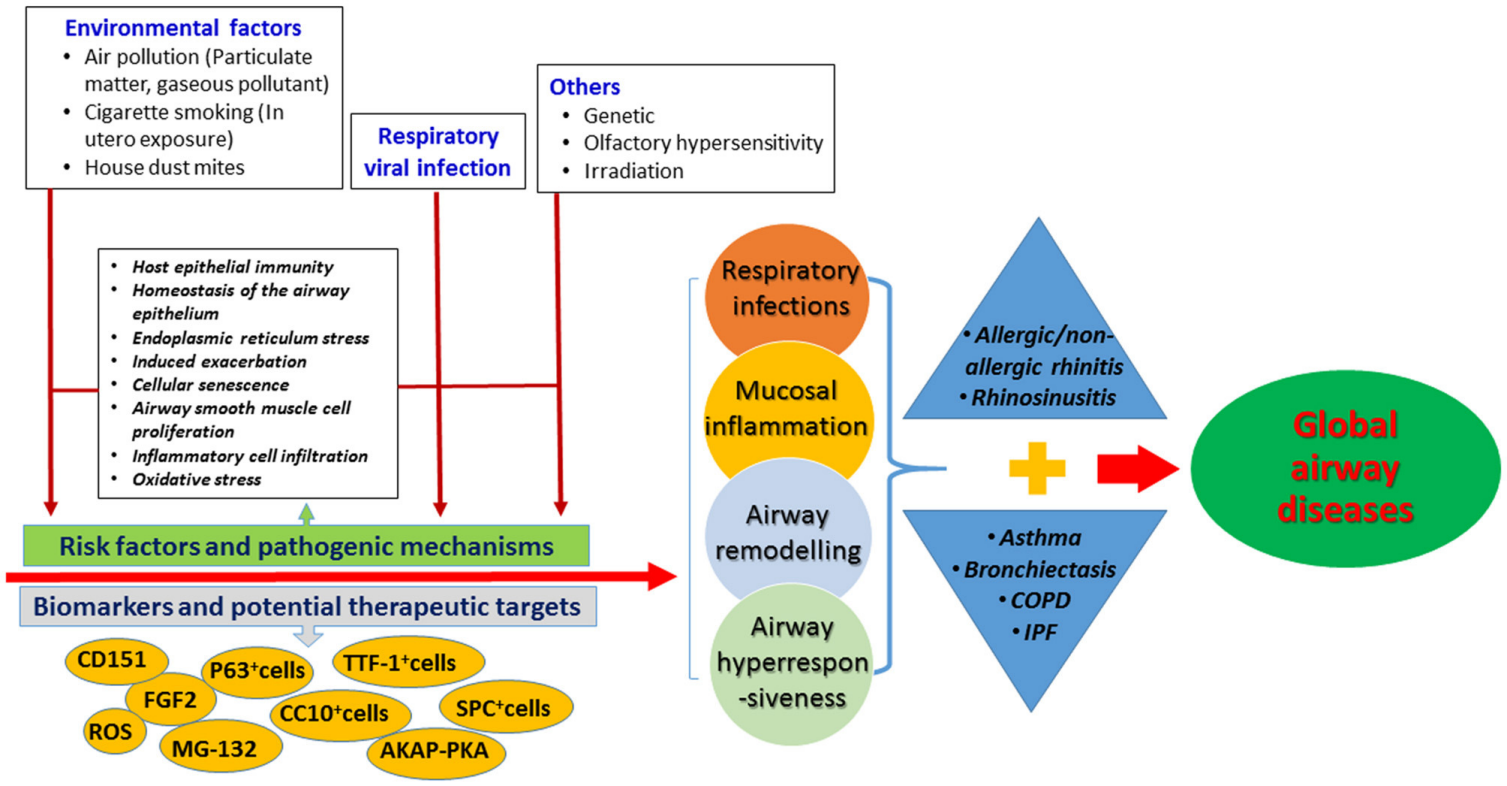
Summary of the risk factors, molecular mechanisms and biomarkers for the global airway diseases in this specific topic. AKAP-PKA, A-kinase anchoring proteins and phosphokinase A; ASM, airway smooth muscle cells; CC10, Club Cell 10 kDa Protein; SPC, Surfactant protein C; TTF-1, thyroid transcription factor-1; CD151, cluster of differentiation 151; FGF2, fibrocyte growth factor-2; ROS, reactive oxygen species.

## Author Contributions

WG, TT, and D-YW: wrote the paper. All authors: critical review and approval.

## Conflict of Interest

The authors declare that the research was conducted in the absence of any commercial or financial relationships that could be construed as a potential conflict of interest.

## Abbreviations

CC10: Club Cell 10 kDa Protein
SPC: Surfactant protein C
TTF-1: thyroid transcription factor-1
CD151: cluster of differentiation 151.
